# Supplementary material for: Cis-Allosteric Regulation of HIV-1 Reverse Transcriptase by Integrase
Source: Viruses. 2022 Dec 21;15(1):31. doi: 10.3390/v15010031 (PMC9864105; doi:10.3390/v15010031)
Supplement: Supplementary file 1 [file viruses-15-00031-s001.zip › Supplementary Figures S1 and S2.pdf]

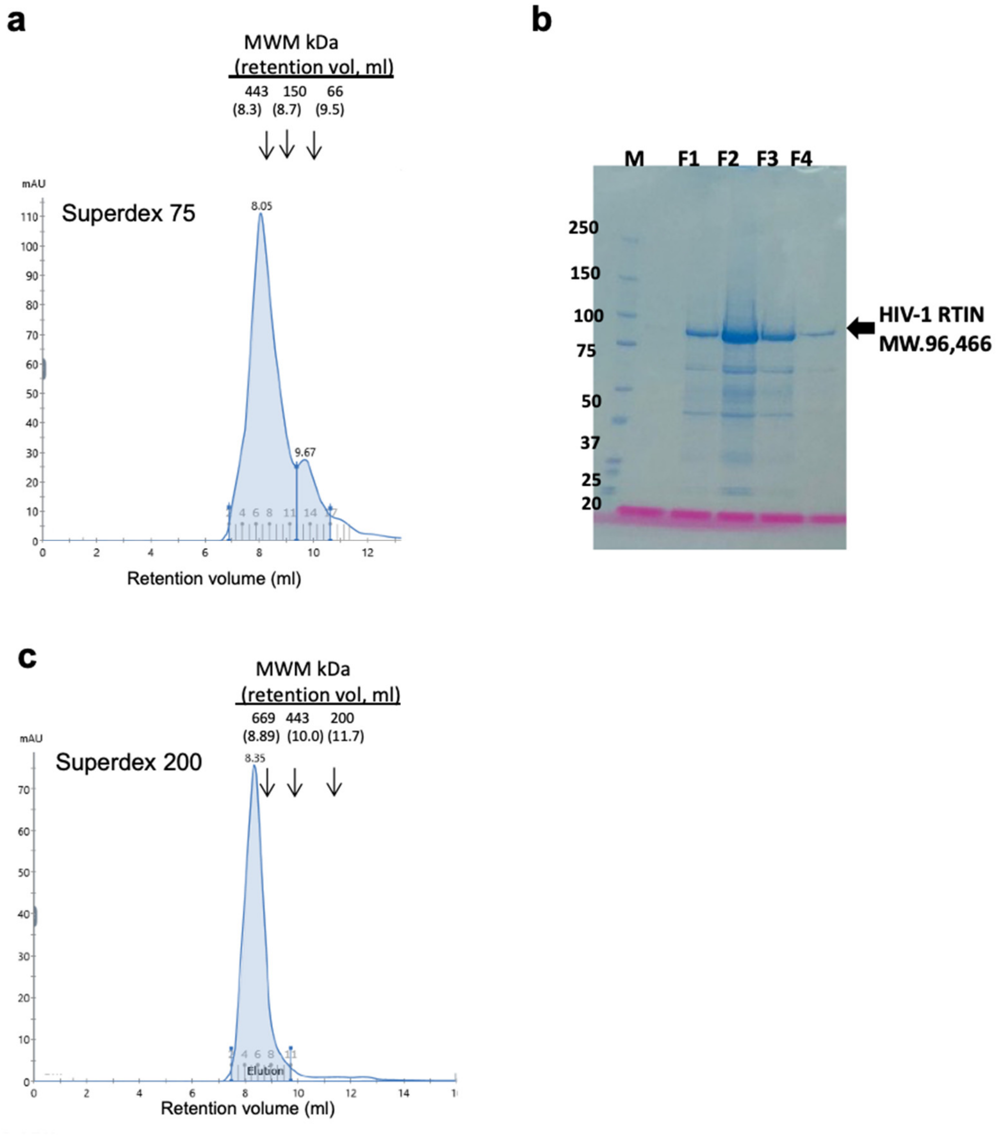

Supplementary Figure S1. SEC profiles of rRTIN. **a.** Purified rRTIN was subjected to a size-exclusion column of Superdex 75 increase 100/300 GL (Cytiva). Retention volume (ml) for each main peak is indicated. To estimate protein size, a molecular weight marker (MWM, Sigma-Aldrich) was also subjected to the same column independently. Locations of MWM proteins are shown on the top with size (kDa) and retention volume (ml). **b.** Fractions of major peaks were subjected to SDS-PAGE followed by staining with Coomassie brilliant blue. Lanes: M (size marker), F1 (fractions of #2-4), F2 (fractions of #5-11), F3 (fractions of #12-14), F4 (fractions of #15-17). Band corresponding to RTIN (molecular weight: 96,466) is indicated. **c.** Major peak fractions (F1-F3) were pooled and subjected to a size-exclusion column of Superdex 200 increase 100/300 GL (Cytiva) as described in **a.**

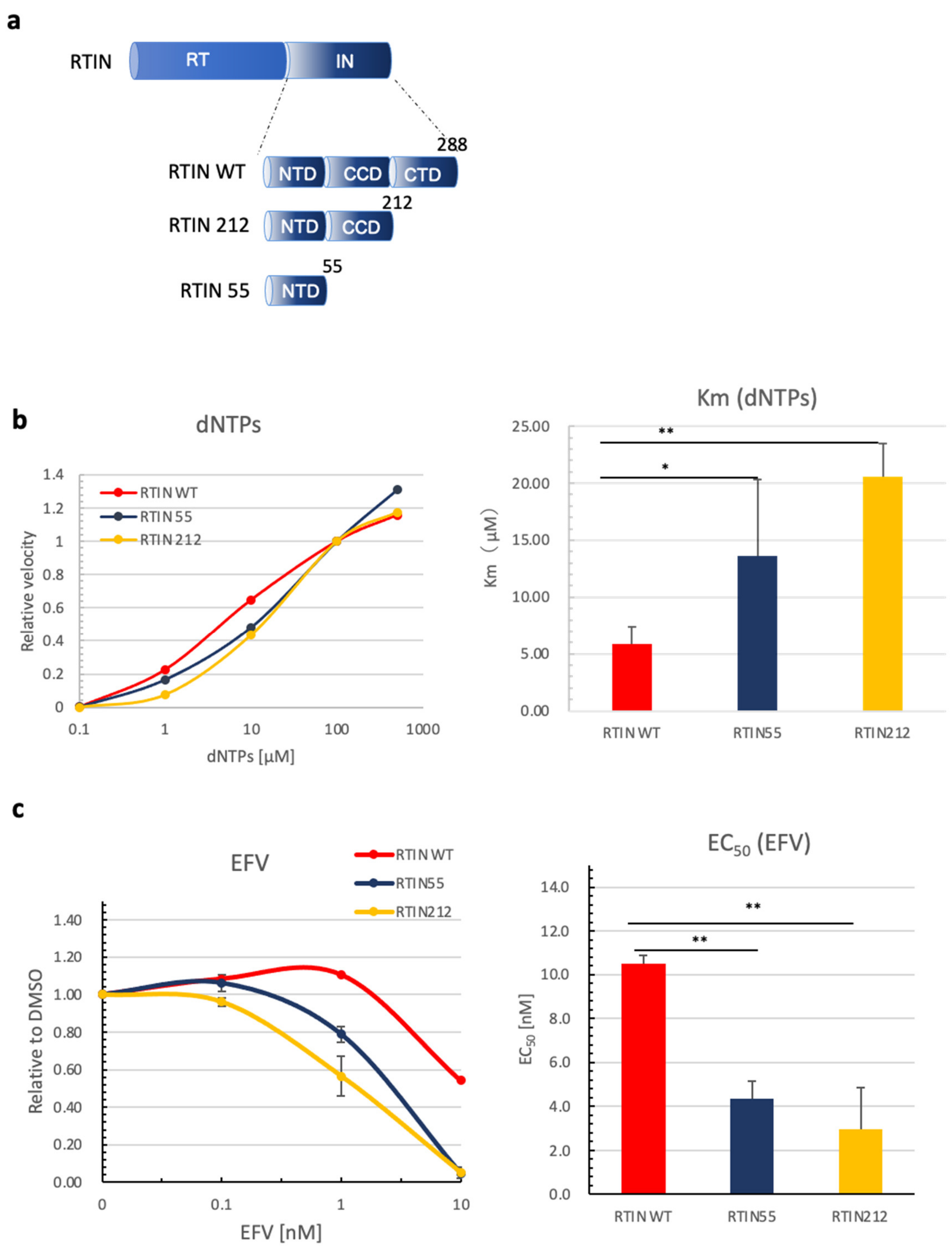

Supplementary Figure S2. Contribution of IN domains on RTIN functions. **a.** rRT carrying IN NTD (residues 1-55, RTIN 55), NTD and CCD (residues 1-212, RTIN 212) or NTD, CCD and CTD (residues 1-288 RTIN WT) are schematically illustrated. **b.** *In vitro* reverse transcription assay was performed in the presence of different concentrations of dNTP. Levels of R/u5 products were measured and are shown as velocity values relative to the level at 100 μM of dNTPs as 1.0 (left). Km to dNTPs for each form of RTIN was calculated as

described in Figure 4 (right). Data are shown with means ± SD (n=3). **c.** *In vitro* reverse transcription assay was performed in the presence of different concentrations of efavirenz (EFV). Levels of R/u5 products were measured and are shown as values relative to the level with solvent (DMSO) only as 1.0 (left). EC_{50} of EFV for each rRTIN was determined by calculating drug concentration to inhibit 50% of RDDP activity in solvent only (right). Data are shown with means ± SD (n=3). Student's *t* analysis showed significant differences between RTIN WT and either RTIN 55 or RTIN 212 (*$p<0.05$, **$p<0.01$).
